# TiO_2_-Based Nanocomposites Thin Film Having Boosted Photocatalytic Activity for Xenobiotics Water Pollution Remediation

**DOI:** 10.3390/nano11020400

**Published:** 2021-02-04

**Authors:** Angelo Nicosia, Fabiana Vento, Gisella Maria Di Mari, Luisa D’Urso, Placido G. Mineo

**Affiliations:** 1 Department of Chemical Sciences and I.N.S.T.M. UdR of Catania, University of Catania, V.le A. Doria 6, I-95125 Catania, Italy; fabiana.vento@phd.unict.it (F.V.); gisella.dimari@dfa.unict.it (G.M.D.M.); ldurso@unict.it (L.D.); 2 Institute for Chemical and Physical Processes CNR-IPCF, Viale F. Stagno d’Alcontres 37, I-98158 Messina, Italy; 3 Institute of Polymers, Composites and Biomaterials CNR-IPCB, Via P. Gaifami 18, I-95126 Catania, Italy

**Keywords:** polymer supported photocatalyst, PMMA, Aeroxide^®^ P25 titanium dioxide nanoparticles, thin film, water remediation, methylene blue, rhodamine B, paraquat, acetaminophen, in situ polymerization

## Abstract

Photocatalytic remediation represents a potential sustainable solution to the abatement of xenobiotic pollutants released within the water environment. Aeroxide^®^ P25 titanium dioxide nanoparticles (TiO_2_ NPs) are well-known as one of the most efficient photocatalysts in several applications, and have also been investigated in water remediation as suspended powder. Recently, their application in the form of thin films has been revealed as a potential alternative to avoid time-consuming filtration processes. Polymers represent suitable substrates to immobilize TiO_2_ NPs, allowing further production of thin films that can be exploited as a photoactive coating for environmental remediation. Nevertheless, the methods adopted to immobilize TiO_2_ NPs on polymer matrix involve time-consuming procedures and the use of several reactants. Here, titanium dioxide-based nanocomposites (NCx) were obtained through a new approach based on Methyl Methacrylate in situ bulk polymerization and were compared with a blended mixture (BL). Their morphology and chemical–physical properties were investigated through Thermogravimetric Analysis (TGA), Differential Scanning Calorimetry (DSC), UV–Vis, and Raman spectroscopies. It was revealed that the in situ approach deeply influences the chemical–physical interactions between the polymer matrix and TiO_2_ NPs. Photocatalytic experiments revealed the boosted photodegradation activity of NCx thin films, induced by the in situ approach. The photodegradation of paraquat and acetaminophen was also ascertained.

## 1. Introduction

Human activities have been deeply influencing the environment, sometimes so intensively that they alter the biosphere by introducing xenobiotic contaminants, especially in water, so determining the Anthropocene era [1,2,3]. Pesticides and drugs represent common pollutants of groundwater due to runoff from the agricultural fields and the release of wastewater within the environment, respectively [4,5]. Thus, current efforts are focused on the development of sustainable systems to pursue environmental remediation, efficiently using renewable energy sources. In this field, photocatalysis represents a challenging solution, and Aeroxide^®^ P25 titanium dioxide nanoparticles (TiO_2_ NPs) are well-known as one of the most efficient photocatalysts in several applications [6,7]. In particular, despite the band gap making it active upon UV irradiation [8] (reducing photonic efficiency when exposed to solar light range radiation), TiO_2_ NPs are largely used for water remediation purposes [7].

In particular, TiO_2_ NPs are commonly applied as water suspended powder (or slurry) [9], having concentrations of about hundreds of mg/L: despite this approach ensuring a high number of photoactive sites and optimizing mass transfer, it also implicates further separation processes. Indeed, it is necessary to avoid any ultrafine material release into the environment, increasing the costs of remediation treatments [10,11]. This semiconductor photocatalyst has also already been used to produce self-cleaning surfaces and/or other value-added materials [12,13,14]. As an example, concrete mixtures with TiO_2_ NPs are used to produce self-cleaning white surfaces and/or depollution cementitious materials [15]. These approaches accept the reduced photonic efficiency of TiO_2_ NPs (under solar radiation) against the ease of practical application; nevertheless, a huge amount of nanomaterial would be hidden in the bulk of the material, preventing its photocatalytic activity.

To enhance the amount of surface-exposed NPs rather than the one embedded within the bulk, TiO_2_-based coatings have been investigated. These could be produced through the deposition of inorganic TiO_2_ NPs [16,17] or polymer-supported TiO_2_ thin films. The latter represent a potential industrial-scale application due to the ease of use in the forming of thin films, and the consequent potential deposition over several substrates.

In the field of water remediation, thin films of polymer-supported photocatalysts also represent a potential alternative to the use of TiO_2_ NPs in slurry form: anchoring NPs onto a polymeric matrix overtakes any expensive and time-consuming filtration processes needed to separate NPs from purified water. Generally, the methods to immobilize TiO_2_ NPs on polymer matrix involve time-consuming procedures and the use of several reactants [18,19,20,21].

An easy method might be to simply blend the polymer with the active photocatalyst [22], even if the instability of the blend’s organic suspension might be a critical point for industrial applications.

As support for photocatalytic NPs, poly(methyl methacrylate) (PMMA) is a suitable candidate due to its stability, filmability, and its transparency to visible light range radiations.

In this communication, we report preliminary results about a new approach based on methyl methacrylate (MMA) in situ bulk polymerization to produce photoactive polymer-supported TiO_2_ NPs with enhanced photocatalytic efficiency. In particular, three PMMA-based nanocomposites containing different amounts of anchored TiO_2_ NPs (NCx) were produced and compared with a PMMA homopolymer and a PMMA-TiO_2_ NPs blend (BL), respectively. The thermal properties were investigated by Thermogravimetric Analysis (TGA) and Differential Scanning Calorimetry (DSC). Both NCx and BL, in the form of thin films, were furtherly characterized by UV–Vis and Raman spectroscopies. The stability of dispersions of nanocomposites in organic solvent was also investigated. The Tauc plot method was applied to estimate the band gap energy of the semiconductor photocatalysts embedded within the polymer matrix. Then, photocatalytic efficiency was determined by applying the thin films in photodegradation experiments of Methylene Blue and Rhodamine B as model dyes, revealing a boosted photocatalytic efficiency of NCx. Finally, the NCx thin films have been employed in the photodegradation of xenobiotic pollutants, in particular acetaminophen and paraquat.

To our knowledge, this is the first case of a synthetic approach that boosts photocatalytic properties without prior functionalization of TiO_2_ NPs.

## 2. Materials and Methods

Methyl methacrylate (MMA), AIBN, THF, Aeroxide^®^ P25 titanium dioxide nanoparticles (21 nm primary particle size (TEM), 35–65 m^2^/g (BET)), Paraquat, Acetaminophen, sulfuric acid (98%), hydrogen peroxide (30%), and LC-MS grade water were purchased from Sigma-Aldrich (Merck Group, Milan, Italy).

### 2.1. Instruments

Thermogravimetric analyses were conducted using a Pyris TGA 7equipped with a TAC 7/DX (Perkin Elmer, Waltham, MA, USA) with a thermal ramp of 10 °C min^−1^, range 50–800 °C, in air (60 mL min^−1^).

Differential scanning calorimetry (DSC) measurements were conducted using a DSC Q20 (TA instruments, New Castle, DE, USA), equipped with a Refrigerant Cooling System, with a heating rate of 10 °C min^−1^, in a specific temperature range and under an anhydrous N_2_ atmosphere (60 mL min^−1^).

UV–Vis spectra were recorded on a Cary60 UV–Vis spectrophotometer (Agilent Technologies, Santa Clara, CA, USA) at 25 ± 0.1 °C, using quartz cuvettes with a 1 cm path length and water (LC-MS grade) as the solvent.

UV–Vis-Diffuse Reflectance (UV–Vis DRS) spectra, in the range of 200–800 nm, were recorded on NC1 and BL films by using a Cary60 UV–Vis spectrophotometer (Agilent Technologies, Santa Clara, CA, USA), and the bandgap energies (E_g_) of the TiO_2_/PMMA samples were estimated by plotting the modified Kubelka–Munk function.

The crystalline phase of TiO_2_ nanoparticles and their dispersion homogeneity in the polymer films were also investigated by analyzing Raman scattering data and Raman imaging maps obtained at 141 cm^−1^ (corresponding to the most intense vibrational signal of the anatase phase). Raman spectra have been excited with a 532 nm laser line of a Coherent Compass Sapphire Laser (Coherent Inc., Santa Clara, CA, USA) mounted on a WITec alpha 300 (Wissenschaftliche Instrumente und Technologie GmbH, Ulm, Germany) confocal Raman apparatus. Spectra have been collected with a 50× objective (spot size of about 2 μm).

### 2.2. Polymethyl Methacrylate and Nanocomposites Synthesis

The poly(methyl methacrylate) (PMMA) was synthesized by means of bulk polymerization. Previously, the methyl methacrylate monomer (MMA) was purified through sodium-sulfate and further basic alumina treatments. Then, the purified MMA (1 g, ~10 mmol) was mixed with the initiator AIBN (17 mg, ~0.1 mmol) within an 8 mL vial, and the mixture was kept in a heating bath at 55 °C for 20 h. Finally, the mixture was dissolved in THF (10 mL) and precipitated in *n*-hexane (60 mL). The polymer was separated through Buchner filtration and dried in a vacuum oven (50 °C, 20 h). The nanocomposites were produced by means of the same conditions. The MMA monomer, after purification, was kept in an 8 mL vial and the requested amount of Aeroxide^®^ P25 titanium dioxide was added. In particular, 50, 12, and 7 mg of TiO_2_ were added to the monomer, to produce the 5%, 1%, and 0.6% PMMA-TiO_2_ nanocomposites, respectively. The mixtures were sonicated for 30 min together with the purified monomer. Then, the AIBN initiator was added, and polymerization was performed as described before. The repeatability of the synthesis procedure has been checked several times.

### 2.3. Production of PMMA-Titanium Dioxide Blend

Briefly, PMMA (100 mg) and TiO_2_ NPs (5 mg) were mixed in THF (5 mL) by using an ultrasonic bath (30 min). Then, the obtained blend, called BL, was dried in a vacuum oven (50 °C, 12 h).

### 2.4. Colorimetric Determination of TiO_2_ Content in the Nanocomposites

Briefly, the nanocomposites in powder form were dispersed into a flask containing concentrated sulfuric acid (15 mL). The mixture was kept under reflux to mineralize the polymeric matrix of the nanocomposites, until the formation of black carbon; then, the solution was maintained for two hours at room temperature, adding 1 mL (per hour) of H_2_O_2_ (30% *v*/*v*) to oxidize the carbon residue and make a complex with the titanium dioxide.

The so obtained non-colored solution was diluted up to 50 mL with water; the addition of water makes the solution turn yellow because of the formation of the titanium(IV)–hydrogen peroxide complex. An excess of 0.5 mL of H_2_O_2_ was then added to be sure of complexing all the titanium ions in solution. The concentration of the complex in solution was finally determined through the titanium(IV)–hydrogen peroxide complex calibration curve (ε = 799 M^−1^cm^−1^).

### 2.5. Thin Film Preparation for UV–Vis Measurements

**Figure 1:**
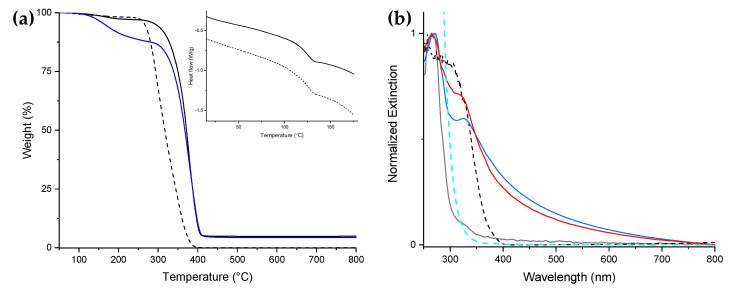
(**a**) TGA traces (air atmosphere) of PMMA (black dashed line), NC1 (black line), and BL (blue line). Inset: DSC traces of NC1 (black line) and PMMA (black dashed line). (**b**) UV–Vis normalized spectra of PMMA (black line), BL (blue line), and NC1 thin films (red line), TiO_2_ powder (black dashed line), and glass (cyan dashed line).

For each of the PMMA, NC1, and Blend, about 1 mg of sample was dissolved in 1:1 (*v*/*v*) CH_2_Cl_2_/Diethyl Ether mixture (300 µL). A few drops of the solution were drop cast onto the wall of a quartz cuvette (1 cm path length). The UV–Vis measurements (Figure 1b) were acquired after thin films dried under N_2_ flux (5 h).

### 2.6. Tauc Plot Method

Diffuse reflectance spectra were recorded and the Kubelka–Munk transformation [23] was applied to convert the diffuse reflectance measurements into the equivalent absorption coefficient.

The obtained absorption values were, in turn, used for the construction of the so-called Tauc plots [24,25,26] and the optical band gap (*E_g_*) values for TiO_2_ were further determined near the absorption edge, using the following expression:

(α*h*ν)^(1/n)^ ∝ (*h*ν − E_g_)

where *α* is the absorption coefficient, *h* is Planck’s constant, and *ν* is the frequency. Depending on the nature of electronic transitions (allowing direct or indirect) in semiconductors, the *n* factor is assumed equal to 1/2 for the direct transitions and 2 for the indirect band gaps.

### 2.7. Photocatalysis Experiments

For each sample, about 15 mg of sample was dissolved in THF (1.5 mL) and deposited onto the inner wall of a 150 mL glass test tube, covering only 180° of the inner circumference (surface area of about 59 cm^2^). The photocatalysis experiments were performed employing an open-wall homemade apparatus (see Section 3), where the test tube was fixed onto a magnetic stirrer, 30 cm away from a solar lamp [OSRAM ultra vitalux (OSRAM Opto Semiconductors GmbH, Regensburg, Germany), Wattage 300 W, Radiated Power: (315–400 nm) 13.6 W, (280–315 nm) 3.0 W, Wavelength range 280–1000 nm] and a fan to cool down the whole system.

For each batch experiment, a volume (100 mL) of dye in water solution, having a concentration suitable to start the experiment from absorbance values of about 0.8 a.u, was treated. The aqueous solutions were used as they are (without any degassing or aerating process).

To equilibrate the dye’s physical absorption onto the polymer thin film, a preliminary dark-storage period of 60 min was performed. Then, the lamp was turned on and the solution continued to be stirred for 120 min more, at a chamber temperature of about 35 ± 1 °C.

Briefly, from the end of the dark-storage time (*t* = 0, *C*_0_), the variations of the dye concentration (*C/C*_0_) in function of the reaction time (Figure 4a,b) were monitored by collecting and analyzing aliquots every 20 min for 2 h (from *C*_0_ to *C*_120_).

In the pseudo-first order photodegradation kinetics, the linear fit slope of the *ln(C/C*_0_*)* vs. time plot represents the kinetic constant (*k*) of the photodegradation.

The photodegradation experiments of Paraquat and Acetaminophen were performed employing the same apparatus and conditions, starting from concentrations of about 80 μM for each pollutant.

## 3. Results

Aiming to develop photoactive coatings suitable for water remediation, poly(methyl methacrylate) (PMMA)-based nanocomposites were synthesized through a new approach based on in situ polymerization with Aeroxide^®^ P25 titanium dioxide nanoparticles (TiO_2_ NPs). Bulk in situ polymerization will ensure the establishing of carbonyl–TiO_2_ NPs interactions, providing the intercalation of the polymer chains within the TiO_2_ NPs porous microstructure [27,28]. To highlight the influence of the polymer on photocatalytic efficiency, the properties of these in situ nanocomposites were compared with those of a blended mixture of the two moieties.

In particular, three PMMA-based nanocomposites containing different amounts (about 5%, 3%, and 0.6% [w]) of anchored TiO_2_ NPs (namely NC1, NC2, and NC3, respectively) were produced and compared with a PMMA homopolymer and a PMMA-TiO_2_ NPs blend containing 5% (w) of TiO_2_ NPs (namely BL).

The degradation temperature of the nanocomposite NC1, including the TiO_2_ NPs (378 °C), was higher than that of PMMA (356 °C) in the TGA measurement (Figure 1a). The DSC traces did not show any significant difference in the glass transition temperatures between NC1 and PMMA (126 and 127 °C, respectively). We confirmed that the temperature range for applying the nanocomposite matches that of the PMMA homopolymer (Figure 1a, inset).

TiO_2_ NPs weight percentage content was determined utilizing a colorimetric technique exploiting the formation of a Ti(IV)–hydrogen peroxide complex (see Section 2.4) [29]. The results are reported in Table 1.

UV–Vis measurements were conducted on thin films of PMMA, NC1, and BL. The PMMA exhibits a narrow absorption centered at 265 nm and could be assumed as transparent in the wavelength range 315–800 nm (Figure 1b, black line). Indeed, the glass test tube used to perform the photocatalytic experiments absorbs light at wavelengths <315 nm (Figure 1b, cyan dashed line), as expected, thus shielding the tube content from the UVB radiations. By comparing the extinction profiles of NC1 and BL (Figure 1b, red and blue continuous lines, respectively), both exhibited TiO_2_ NPs presence: NC1 shows a narrow and intense signal centered at 320 nm, while BL shows a lower intensity broader signal centered at 327 nm. The UV blue shift is usually attributed to the decrease in particle size due to the quantum confinement effect [30,31]. Taking into account also the narrowing of the band and the higher intensity of the NC1 extinction signal, these data suggest a better dispersion of TiO_2_ NPs within the PMMA matrix than that of BL, improving the photonic efficiency of the photocatalyst.

**Figure 2:**
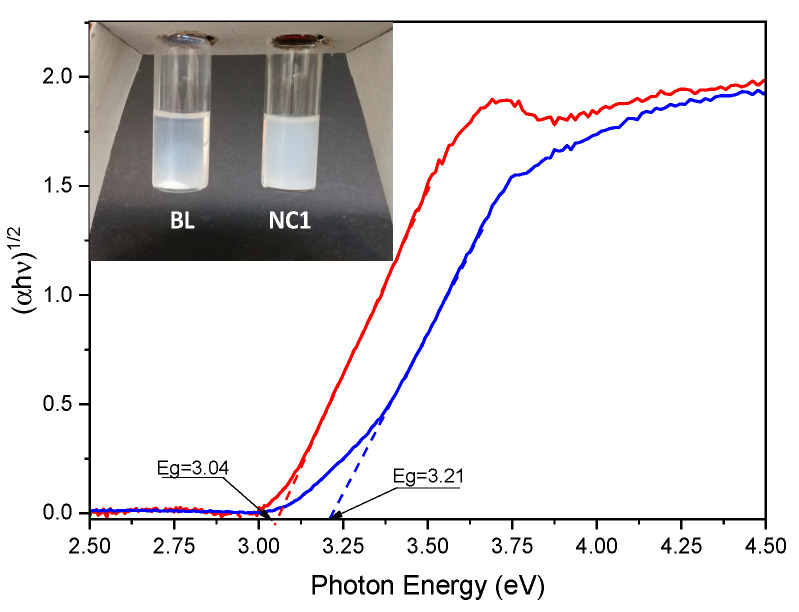
Calculation of the optical band gap of NC1 (**blue**) and BL (**red**) through the Tauc plot method. Inset: digital image of BL and NC1 THF solutions after 24 h storage.

Moreover, by comparing the stability of the solutions of NC1 and BL in THF (7.5 mg/mL), the BL solution shows precipitate after 24 h (Figure 2, inset). On the contrary, the NC1 solution also does not exhibit any precipitate after months of storage, as expected from the strong interaction between TiO_2_ NPs and the polymeric matrix ensured by the in situ synthetic approach. Indeed, in this case, the polymerization will also occur inside TiO_2_ NPs pores; on the other hand, the polymer only wraps NPs in a blended mixture.

In order to investigate the optical band gap of produced BL and NC1 films, diffuse reflectance spectra were recorded and the Kubelka–Munk transformation [23] was applied to convert the diffuse reflectance measurements into the equivalent absorption coefficient (see Section 2.6); then, the obtained absorption values were in turn used for the construction of the so-called Tauc plots [24,25,26] (Figure 2). Therefore, by applying the Tauc approach and by plotting (*αhν*)^1*/n*^ versus the photon energy *(hν*) for BL and NC1 films, a linear relationship was obtained only when indirect allowed transitions (*n* = 2) were considered, obtaining an extrapolated energy bandgap (*E_g_*) value of about 3.21 eV for the NC1 sample and of 3.04 eV for BL.

It is necessary to highlight that, although these values are acceptable and in accordance with the literature values [32,33,34] reported for rutile (3.02–3.24 eV) and anatase (3.23–3.59 eV), the application of the Tauc method to heterogeneous systems, as in our case composed of mixed semiconductor phases in a polymer, is critical and can lead to an inaccurate estimation of *E_g_*. In these cases, as reported in the literature [35,36,37], the absorption coefficient exhibits more complex signals than a crystalline homogenous semiconductor phase and it is, therefore, difficult to discriminate the transition mode and hence, to extrapolate the *E_g_* value, simply considering the Tauc’s approach.

**Figure 3:**
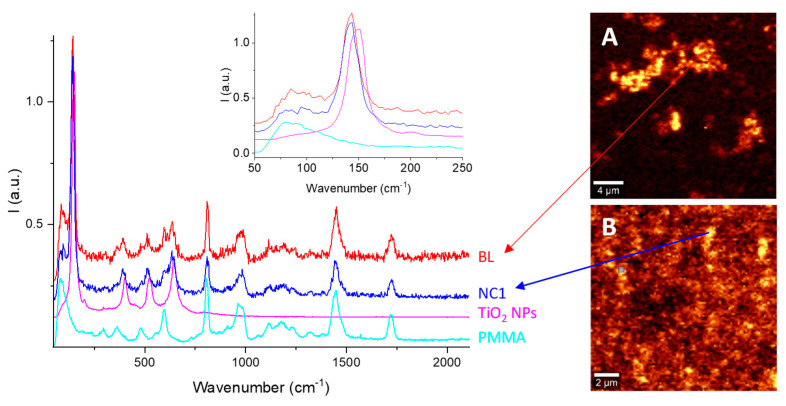
Raman spectra of PMMA (cyan), TiO_2_ NPs powder (magenta), NC1 (blue), BL (red), and Raman imaging map of NC1 thin film (**A**, 10 × 10 µm) and BL thin film (**B**, 20 × 20 µm). Brighter spots indicate TiO_2_ NPs Raman scattering at 141 cm^−1^. Inset: focus on the region of the E_g_(1) vibration mode.

The Raman spectrum of the bare TiO_2_ NPs (Figure 3, magenta line) shows bands at 148, 200, 402, 523, and 644 cm^−1^, attributed to the E_g_(1), E_g_(2), B_1g_(1), B_1g_(2) + A_1g_, and E_g_(3) vibration mode typical of the anatase phase [38]. As expected, both the BL and NC1 spectra exhibited the overlap of the PMMA and TiO_2_ Raman bands. Nevertheless, in both cases (Figure 3, inset), slightly red-shifted TiO_2_-related bands are shown: from 148 cm^−1^ up to 142 cm^−1^. Since the red-shift of Raman signals has been reported as an effect of the formation of oxygen vacancies in the Ti^IV^ lattice [39], it suggests a “doping effect” of the polymer on the titanium dioxide.

Despite their negligible differences in Raman shifts, Raman imaging maps obtained using the signal at 141 cm^−1^ showed a higher homogeneity of TiO_2_ NPs dispersion within NC1 than within BL thin film (Figure 3A,B, respectively), confirming the UV–Vis results.

**Figure 4:**
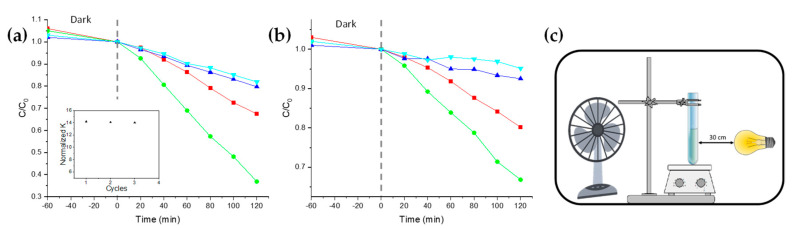
(**a**) Methylene Blue concentration ratio (C/C_0_) variations in function of the irradiation time in the presence of the thin films of NC1 (green), NC2 (blue), NC3 (cyan), and BL (red). Inset: Rate constant (normalized by weight) during the reuse of the NC1 thin film with MB. (**b**) The same plot is reported for Rhodamine B photodegradation. (**c**) Simplified scheme of the open-wall homemade apparatus employed in these experiments.

The photocatalytic efficiency of both nanocomposites and BL was determined through photocatalytic degradation experiments involving Methylene Blue (MB) and Rhodamine B (RhodB), performed with a suitable solar lamp in an open-wall homemade apparatus (Figure 4c). The catalyst samples were employed as thin films (area of about 59 cm^2^) deposited onto the glass tube inner surface (see Section 2.7).

The variations of dye concentration (*C/C*_0_) in function of the reaction time were monitored. Since both MB and RhodB are known to have pseudo-first order photodegradation kinetics [40,41], the linear fit slope of the *ln(C/C*_0_*)* vs. time plot represents the kinetic constant (*k*) of the photodegradation.

Particular care was taken to avoid any overestimation of the photocatalytic activity: the absence of any photolysis process of both MB and RhodB, and also the negligibility of the potential adsorption of dye onto the polymer film, were confirmed by applying the same experimental conditions over dye solutions with and without a PMMA homopolymer film.

By comparing the calculated *k* of the nanocomposite NC1 with one of BL, the improved photocatalytic activity of the nanocomposites is evident (Table 1). In particular, NC1 is about 2.8 times more active in the presence of MB (Figure 4a), and about 2 times in the presence of RhodB (Figure 4b). An obvious *k* decrease is exhibited for the samples NC2 and NC3, due to the lower TiO_2_ NPs amount loaded within the nanocomposites (Figure 4a,b, blue and cyan lines, respectively).

For a better comparison of the photocatalytic efficiency of the thin films employed during the experiments, the mass of embedded catalyst by normalizing the *k* must be taken into account [42]. The *normalized k* was here defined as *k/grams TiO_2_*, where TiO_2_ NPs content was previously determined via a colorimetric method.

The outcome (Table 1) shows that the catalytic efficiency of nanocomposites is approximately constant despite the reduction in TiO_2_ NPs content (with both the dyes), and still higher than BL. The standard errors of *normalized k*, calculated on the photocatalytic experiments performed in triplicate, reported in Table 1, result in accordance with the literature for dyes photodegradation [40].

The reusability of the thin films was confirmed by the reuse of the same film for up to three photodegradation cycles of MB solutions. In order to avoid the presence of any traces of the dye or some intermediate species formed and remaining adsorbed on the film during the photodegradation process, after each cycle, the test tube containing the film was copiously washed under solar lamp irradiation, and refilled with fresh MB solutions. The experiments (Figure 4a, inset) evidenced a decrease in the *normalized k* of about 1.01% for each photocatalytic cycle.

**Figure 5:**
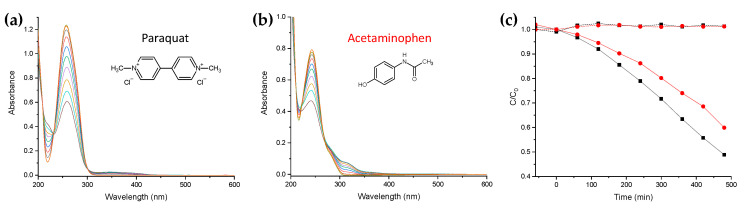
UV–vis spectra acquired during Paraquat (**a**) and Acetaminophen (**b**) photodegradation, with the related molecular structures. (**c**) Variations of Paraquat (Black) and Acetaminophen (Red) concentration ratio (C/C_0_) in function of the irradiation time in the presence of the thin films of NC1. The dashed lines represent the related photolysis.

To verify the NC1 thin films’ photocatalytic efficiency against herbicide and pharmaceutical pollution, the NC1 was also applied in photodegradation experiments of Paraquat and Acetaminophen (or Paracetamol, Figure 5) water solutions, respectively. The negligibility of the photolysis event was ascertained prior. The experiments show degradation of about 51% for Paraquat and 40% for Paracetamol (Figure 5) after 480 min.

Noteworthy is that the amount of active photocatalyst (embedded within the in situ polymerized matrix) adopted here was 6.5 mg per liter of treated polluted water, in contrast with the literature-reported photo-remediation processes involving hundreds of milligrams of TiO_2_ NPs (in the form of slurry) per liter of polluted water [43]. Therefore, by the use of NC1 thin films, the treated water does not need any expensive further filtration treatment to separate the photocatalyst.

## 4. Discussion

The in situ polymerization approach provides huge differences in morphological and functional properties compared to the blended mixture. Despite the NC1 and BL showing negligible variations in degradation temperature as well as glass transition temperature, the spectroscopic properties are deeply modified by the approach proposed here. Indeed, UV–Vis and Raman investigations revealed that the NC1 exhibits a more homogeneous dispersion of TiO_2_ NPs within the PMMA matrix. Moreover, the longer stability of the NC1 organic dispersion confirms the occurrence of strong interactions between polymer chains and inorganic nanoparticles.

The photocatalytic efficiencies of the nanocomposites and the blended mixture were investigated using them in the form of thin films. By means of the model dyes MB and RhodB, the NC1 revealed itself as 2.8 times more active against MB, and about 2 times against RhodB. Dark adsorption data revealed the negligibility of the adsorption phenomenon onto the PMMA substrate, thus suggesting that the dye adsorption phenomenon is due to the availability of TiO_2_ NPs on the surface of the nanocomposites.

The repeatability tests evidenced a loss of about 3% thin film photocatalytic efficiency for each cycle, suggesting the negligibility of active photocatalyst leaching because this event would have stronger negative influences towards photocatalytic efficiency. The same considerations could be applied to the occurrence of nanocomposites’ self-oxidation processes.

The photocatalytic efficiency of the NC1 against organic pollutants was also verified. A photocatalytic abatement of about 51% for Paraquat and 40% for Paracetamol after 480 min was evidenced. Taking into account the variations of concentration ratio (C/C_0_) in function of the irradiation time, as a result of the adsorption of pollutants onto the TiO_2_ surface (shown in Figure 5c), in the first stage, a pre-photoactivation of the nanocomposite/pollutant/water system could occur, leading to the active species that determine the degradation observed in the second stage.

To explain the highest photocatalytic activity of the NCx nanocomposites compared to the blended mixture, it is necessary to focus on both physical and chemical–physical contributions. The physical contribution arises from the better dispersion with reduced aggregation phenomenon of nanoparticles in the NCx polymeric matrix with respect to BL: it makes more photoactive sites available [44], and avoids the decrease in reactive oxygen species generation (which is typical of aggregation phenomena) [45].

Another fundamental contribution to boost the photocatalytic activity of NCx could arise from the differences between organic and inorganic moieties in terms of both refractive index and dielectric coefficient, which are enhanced by the in situ approach. The intercalation of the monomer within NPs pores and the consequent in situ polymerization produce deep chemical–physical interactions, determining a local waveguide effect of the light based on the different refractive index of the moieties [46]. It enhances the light propagation within the photocatalyst nanoparticles and increases the extinction intensity of NC1 compared to BL, as confirmed by UV–Vis measurements.

Another phenomenon should result from the different dielectric coefficients of PMMA and TiO_2_ NPs. Indeed, a strong electronic charge interaction will occur at their interface, producing an electric dipole layer that accelerates the separation of the charges [47]. Such an effect would be reflected in the enhanced photocatalytic efficiency of NC1, thanks to the larger interface area ensured by the in situ approach (in contrast with the blending process).

As expected, by comparing the photodegradation efficiencies of NCx with bare TiO_2_ NPs (employed in the slurry form), the NCx exhibit *normalized k* values almost half that of TiO_2_ NPs (by using the same experimental setup, data not reported for brevity). Nevertheless, the use of NPs slurry results positive to improve the surface area exposed to the dye solution, but must face the issue of nanoparticles separation from the depolluted water. Such an issue is avoided if NCx thin films are employed.

The data reported here bring out the potentiality of the in situ polymerization approach in large-scale applications. From an industrial point of view, this approach candidates itself as a potential alternative to the blended materials because the stability of the nanocomposites organic solutions makes the storage/handling/application methods and the subsequent industrial processes (i.e., spray and/or dip and/or brush coating) easier. The nanocomposites obtained by in situ polymerization are also potentially suitable as a component of a recent photocatalyst panel [48].

Furthermore, here it is demonstrated that the in situ approach enhances by two/three times (depending on the pollutant) the photocatalytic efficiency of the polymer-supported TiO_2_ NPs. The photodegradation activity against xenobiotic pollutants ensures the feasibility of these nanocomposites in water remediation applications.

## Figures and Tables

**Table 1:** Kinetics calculations related to MB and RhodB photodegradation employing different thin films: ^a^ kinetic constant; ^b^ nanocomposite’s TiO_2_ weight % content determined via colorimetric method; ^c^ kinetic constant *k* normalized by weight of TiO_2_ (standard errors are reported in brackets).

Dye	Sample	^a^*k* (min^−1^)	^b^ TiO_2_ (% w)	^c^ Normalized *k* (min^−1^ g^−1^)
Methylene Blue	BL	3.43	5	4.51 (0.26)
	NC1	9.62	4.34	14.2 (0.83)
	NC2	1.89	0.92	13.44 (0.76)
	NC3	1.67	0.69	15.75 (0.89)
Rhodamine B	BL	1.88	5	2.47 (0.16)
	NC1	3.70	4.34	5.61 (0.34)
	NC2	0.63	0.92	4.55 (0.27)
	NC3	0.33	0.69	3.25 (0.20)

## Data Availability

Data is not available due to further project advancement.
